# The Development of Fe_3_O_4_-Monolithic Resorcinol-Formaldehyde Carbon Xerogels Using Ultrasonic-Assisted Synthesis for Arsenic Removal of Drinking Water

**DOI:** 10.3390/gels9080618

**Published:** 2023-07-30

**Authors:** Sasirot Khamkure, Prócoro Gamero-Melo, Sofía Esperanza Garrido-Hoyos, Audberto Reyes-Rosas, Daniella-Esperanza Pacheco-Catalán, Arely Monserrat López-Martínez

**Affiliations:** 1 Postgraduate Department, CONAHCYT-Mexican Institute of Water Technology, Jiutepec 62550, Mexico; 2 Sustainability of Natural Resources and Energy, Cinvestav Saltillo, Ramos Arizpe 25900, Mexico; procoro.gamero@cinvestav.edu.mx (P.G.-M.); arely.lopez@cinvestav.edu.mx (A.M.L.-M.); 3 Postgraduate Department, Mexican Institute of Water Technology, Jiutepec 62550, Mexico; sgarrido@tlaloc.imta.mx; 4 Department of Bioscience and Agrotechnology, Research Center of Applied Chemistry, Saltillo 25294, Mexico; audberto.reyes@ciqa.edu.mx; 5 Renewable Energy Unit, Yucatan Scientific Research Center, Merida 97302, Mexico; dpacheco@cicy.mx

**Keywords:** adsorption, arsenate and arsenite, carbon xerogels, resorcinol-formaldehyde, sonication

## Abstract

Inorganic arsenic in drinking water from groundwater sources is one of the potential causes of arsenic-contaminated environments, and it is highly toxic to human health even at low concentrations. The purpose of this study was to develop a magnetic adsorbent capable of removing arsenic from water. Fe_3_O_4_-monolithic resorcinol-formaldehyde carbon xerogels are a type of porous material that forms when resorcinol and formaldehyde (RF) react to form a polymer network, which is then cross-linked with magnetite. Sonication-assisted direct and indirect methods were investigated for loading Fe_3_O_4_ and achieving optimal mixing and dispersion of Fe_3_O_4_ in the RF solution. Variations of the molar ratios of the catalyst (R/C = 50, 100, 150, and 200), water (R/W = 0.04 and 0.05), and Fe_3_O_4_ (M/R = 0.01, 0.03, 0.05, 0.1, 0.15, and 0.2), and thermal treatment were applied to evaluate their textural properties and adsorption capacities. Magnetic carbon xerogel monoliths (MXRF600) using indirect sonication were pyrolyzed at 600 °C for 6 h with a nitrogen gas flow in the tube furnace. Nanoporous carbon xerogels with a high surface area (292 m^2^/g) and magnetic properties were obtained. The maximum monolayer adsorption capacity of As(III) and As(V) was 694.3 µg/g and 1720.3 µg/g, respectively. The incorporation of magnetite in the xerogel structure was physical, without participation in the polycondensation reaction, as confirmed by XRD, FTIR, and SEM analysis. Therefore, Fe_3_O_4_-monolithic resorcinol-formaldehyde carbon xerogels were developed as a potential adsorbent for the effective removal of arsenic with low and high ranges of As(III) and As(V) concentrations from groundwater.

## 1. Introduction

A current environmental and human health problem is the availability of water due to the increasing demand and contamination of drinking water sources. Most of the accessible drinking water is found in aquifers, which are underground reservoirs of water. However, the presence of contaminants such as arsenic, which naturally occur in the environment due to geological factors, can migrate into groundwater through weathering processes.

Inorganic arsenic (As) is a well-known carcinogenic element and one of the most significant chemical pollutants worldwide, found in several countries across the globe. The Agency for Toxic Substances and Disease Registry (ATSDR) has ranked arsenic as the top substance with potential risks to public health on a global scale [1]. The World Health Organization (WHO) recommends a guideline value of 10 μg/L for arsenic concentrations in drinking water, which is also considered acceptable by the United States Environmental Protection Agency (EPA) [2,3]. In Mexico, the NOM-127-SSA1-2021, “Environmental Health, Water for Human Use and Consumption—Permissible Limits of Quality and Treatments for Water Purification”, sets an allowable limit of 10 μg/L for arsenic in drinking water [4]. Groundwater contamination by arsenic affects millions of people in various countries, including the United States, Argentina, Australia, Bangladesh, Cambodia, Chile, China, India, Laos, Myanmar, Mexico, Pakistan, Taiwan, Thailand, and Vietnam [5,6,7].

Arsenic contamination in drinking water is a serious problem in Mexico. The levels of arsenic in drinking water in some regions of the country exceed the recommended limit of 10 µg/L. Many people in Mexico are at risk due to consuming water with elevated arsenic levels. children are at risk of developing serious health problems as a result of their exposure to arsenic [8]. The concentrations of arsenic in water samples from Chihuahua ranged from 0.1 to 419.8 µg/L, which is associated with adverse health effects [9]. Groundwater background values in Guanajuato State were evaluated, and the arsenic values from sample wells were in the range of 0.068–0.777 mg/L. These values are due to geogenic sources containing volcanic rocks, specifically rhyolites, which have a presence of As and F in the hot deep flow [10]. Three Yaqui villages in southern Sonora, Mexico, have been studied for arsenic exposure through drinking water. The range of arsenic concentration in these villages was 11.8–70.01 μg/L, and it has been associated with lung function and inflammation, as well as respiratory infections in children [11].

Arsenic can exist in various oxidation states, but in natural water sources, it is predominantly found in its inorganic forms as trivalent arsenite (As(III)) or pentavalent arsenate (As(V)) oxyanions. The presence of arsenic-contaminated water that is used for drinking, food preparation, and agricultural irrigation poses a significant threat to public health. Prolonged exposure to arsenic through the ingestion of contaminated food and water can lead to the development of cancer and skin lesions [12]. Arsenic has been linked to various diseases affecting the cardiovascular, liver, neurological, immune, endocrine systems, as well as the skin. It has also been associated with diabetes and various types of cancer, such as skin, liver, lung, and bladder cancer, due to its absorption through the gastrointestinal tract, skin, and respiratory system [13]. Furthermore, elevated levels of arsenic in drinking water have been associated with an increased risk of myocardial infarction [14], adverse effects on fertility in women [15,16], and negative consequences for fetal development during pregnancy [17].

The processes and technologies for the removal of arsenic from water that are known currently are oxidation, precipitation, coagulation and softening with lime, reverse osmosis, microfiltration, nanofiltration, adsorption, biological treatments, phytoremediation, electrodialysis, and electrokinetics, among others [18,19,20]. Among these methods, the application of adsorption is a promising technique and has been widely extended in the treatment of water and wastewater due to its high efficiency, affordability, ease of design, operation, handling, and maintenance, and the variety of adsorbent materials that can be regenerated and reused. Furthermore, no additional chemicals are needed in the operation, and there is no production of sludge or generation of toxic byproducts [7,21].

Some emerging arsenic adsorbents are chemically modified zeolites [22], zeolitic imidazole frameworks [23], lanthanum hydroxide–doped graphene oxide biopolymer foam [24], metal–organic framework-based composite materials [25], and jarosites [26]. The vast majority of reported adsorbents are micro or nanometer-size powders; although some of these materials have a high adsorption capacity for micropollutants from water, their application at the pilot plant or industrial level is limited by the difficulty of separating the adsorbent from the treated water.

Iron-based adsorbents are excellent adsorbents for removing arsenic from water. Magnetite (Fe_3_O_4_) is one of the most well-known iron oxides/hydroxides due to its strong affinity for arsenic and ease of accessibility [27]. Iron-based adsorbents are non-toxic, low cost, and easily accessible in large quantities and offer promising results for arsenic removal from water [28,29]. 

The gels are mesoporous materials with texture, mechanical resistance, and chemical stability. They can be controlled and designed according to the variation of the synthesis and processing conditions. Gels are formed by the addition and/or polycondensation of a low molecular weight oligomer in an aqueous or alcoholic solution. First, a “sol” is formed, a colloidal solution of solid particles that grow and coalesce to the gel point, making the sol-gel transition at which the viscosity of the medium changes. The formed wet gel behaves like a giant molecule of equal size to the container where it is prepared. The gel progressively strengthens as residual unreacted oligomers bind to the developing network. This phenomenon is called aging or curing, and it allows favorable conditions for drying the gel with the least number of breaks in the structure formed. In other words, the gel is composed of a continuous solid skeleton formed by chains of monomer particles arranged in a pearl necklace that is immersed in a continuous liquid phase. By removing the liquid from the wet gel, a large pore volume can be obtained [30]. The solvent that saturates the pores can be evacuated by three following drying methods: subcritical, supercritical, and cryogenic. Subcritical or conventional drying under atmospheric conditions can generate drastic changes in the surface tension of the solvent once the vapor–liquid interface is formed, this difference between the surface tension of the coexisting vapor and liquid phases produces collapses in the pore structure of the gels. The result is a dense polymer called xerogel. A specific application of resorcinol formaldehyde (RF) gel, including the doping of RF gels with metals or metal oxides, is found in the removal of contaminants from drinking water and wastewater [31,32,33,34]. 

Ultrasound technology has been utilized for the synthesis of various materials, including nanoparticles, and has found numerous applications such as homogenizing, emulsifying, dispersing, deagglomeration, sonochemistry, and sono-catalysis. The effects of sonication on agglomeration, metal release, zeta potential, and the administered dose were evaluated using probe sonication for the synthesis of non-functionalized nanoparticles such as copper, aluminum, manganese, and zinc oxide [35]. The results showed that sonication can be used to control the size and morphology of nanoparticles, as well as to improve their dispersibility and zeta potential. Iron(III) trimesate xerogel was prepared using ultrasonic irradiation within a short time of 10 to 20 min and a low pH solution [36]. This method produced a product with a high specific surface area of 1042 m^2^/g. The high specific surface area of the xerogel was attributed to the formation of a porous structure during the ultrasonic irradiation process. Furthermore, silica xerogels were prepared using the sol-gel method with ultrasonic treatment to accelerate aging and hydrophobic treatment [37]. The effect of ultrasonic frequency, specifically 100 kHz and 500 kHz, on the structure was investigated. It was found that 500 kHz accelerated the aging reaction, facilitated hydrophobization, and rapidly suppressed gel shrinkage. These studies demonstrate the potential of ultrasound technology for the synthesis of various materials with desired properties. Ultrasound can be used to control the size, morphology, dispersibility, zeta potential, and aging of nanoparticles. It can also be used to accelerate the formation of porous structures and to improve the hydrophobicity of materials. Ultrasonic technology can be considered an environmentally friendly application because it reduces processing time, increases cost efficiency, simplifies manipulation, enhances the purity of the final product, and lowers energy consumption [32,34].

Regarding the theoretical knowledge of resorcinol-formaldehyde xerogels and the applications of iron-based adsorbents such as Fe_3_O_4_ for arsenic removal, this study applied the ultrasonic-assisted synthesis of carbon xerogels to evaluate the effect of Fe_3_O_4_ loading through both direct and indirect methods on the removal of arsenic species in groundwater. The novelty of this work is a Fe_3_O_4_-monolithic resorcinol-formaldehyde carbon xerogel that, due to its chemical composition and ordered porous structure, is capable of removing arsenite and arsenate ions present in groundwater. Moreover, due to its magnetic properties, it is possible to easily recover it from the treated water.

This research focuses on generating magnetic carbon xerogels with morphological, magnetic, textural, and physical–chemical properties in the form of pellets, which makes them reusable. These materials have the capability to adsorb arsenates and arsenites. The synthesis procedure was developed considering the effect of Fe_3_O_4_ loading via ultrasonic methods, both direct and indirect, while varying the molar ratios of Fe, catalyst, and water. The arsenic adsorption test was conducted in batch, and the synthesized materials were characterized using various analytical techniques before and after the adsorption of arsenic. The intended purpose of this work is for the adsorbent materials produced to serve as viable alternatives within the technological advancements for water remediation. The magnetic properties of carbon xerogels facilitate the separation, reuse, regeneration, and recycling of the adsorbents so that their useful life is extended. The efficient separation of aged adsorbents also facilitates the recovery and final disposal of contaminants and strengthens the environmental sustainability of the water purification process.

The experimental reproducibility of Fe_3_O_4_-monolithic resorcinol-formaldehyde carbon xerogels involves several challenges, including the composition of the starting materials (variation in molar ratios), the homogeneity of dispersion (direct and indirect ultrasonication methods), the sonication conditions (power output, duration, and frequency), the gelation and curing conditions, and the post-synthesis treatments (pyrolysis). Controlling these parameters consistently across different experiments can be challenging, and this can affect the properties of the resulting monolithic carbon xerogels. Subsequently, the adsorption capacity of the final material was improved with increasing the initial concentration and the adsorption affinity for arsenic species.

The environmental sustainability of the arsenic adsorption process using Fe_3_O_4_-monolithic resorcinol-formaldehyde carbon xerogels can be evaluated through methodologies such as the lifecycle, planetary boundaries, and sustainable development goals [38]. However, applying these methodologies and their indicators is beyond the scope of this article.

## 2. Results and Discussion

### 2.1. Fe_3_O_4_-Monolithic Resorcinol-Formaldehyde Xerogels: Effect of Loading of Magnetite with Indirect and Direct Sonication, and Modification of Catalyst

This study focuses on the development and initial preparation of monolithic xerogels with magnetic properties using magnetite (Fe_3_O_4_, Lanxess) as an adsorbent material for water treatment. The magnetic xerogel monoliths (MCs and MXs) were synthesized through the sol-gel polymerization of resorcinol and formaldehyde (RF) with sodium carbonate (C) as a catalyst, employing indirect and direct sonication, respectively. The effect of varying the molar ratios of resorcinol/catalyst was evaluated to obtain the high adsorption capacity in the arsenate adsorption.

#### 2.1.1. Characterization of MCs and MXs

**Figure 1:**
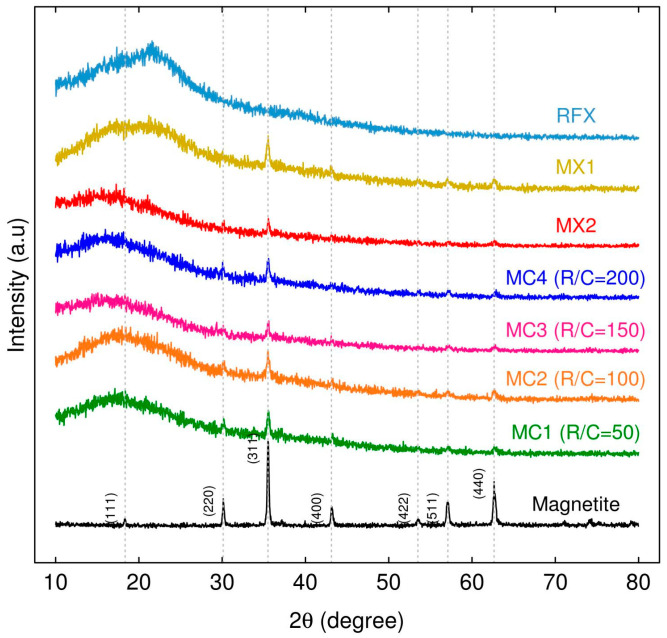
Powder X-ray diffraction patterns of the xerogels (RFX), Fe_3_O_4_-monolithic resorcinol-formaldehyde xerogels synthesized through direct sonication (MX1 and MX2), and indirect sonication with different R/C (MC1-MC4), along with their corresponding JCPDS card assignments.

To identify the phases in the xerogels, XRD analysis was carried out. XRD pattern of RFX revealed the presence of both crystalline and amorphous phases as shown in Figure 1, which is similar to the pattern reported by [39,40].

The XRD patterns of magnetic xerogels prepared using direct and indirect sonication methods and different R/C ratios (Figure 1) were found to be similar, with diffraction peaks at 2 θ values of 18°, 30°, 35.5°, 43°, 57°, and 62° corresponding to the crystallographic planes of magnetite 111, 220, 311, 400, 511, and 440, as reported in the ICCD card number 00-01900629. These findings align with the research of [41]. The percentage of crystalline phase for RFX, MX1, and MX2 was 10.54%, 12.45%, and 10.51%, respectively. Meanwhile, the percentage of crystalline phase for MC1-MC4 ranged from 10.51% to 12.64%. The percentage of crystalline phase for magnetic xerogels prepared using direct and indirect methods (MX1 and MC1) at the same molar ratios and gelation process was approximately 12%.

The crystal size of the magnetic xerogels was determined by calculating the X-ray diffraction peak widths using Bragg’s law and Debye Scherrer equation, as described by [42].

D = Kλ/βCosθ,(1)

where D is the crystalline size, K denotes represents the Scherrer constant (0.98), λ represents the X-rays wavelength (1.54178 Å), β denotes the full width at half maximum (FWHM) and θ is the Bragg diffraction angle (radians).

Table 1 shows the average crystalline sizes of MC50, MC100, and MC200 were in the range of 22.94–25.88 nm. Additionally, their values of β (0.32–0.36 radians) and θ (35.52–35.53 radians) were similar. However, the crystallinity of particle of MC200 (R/C = 200) was higher than MC50 (R/C = 50) and MC100 (R/C = 100). This indicates that increasing the R/C ratio can result in increased crystallinity, which is similar to the results obtained by [43].

**Figure 2:**
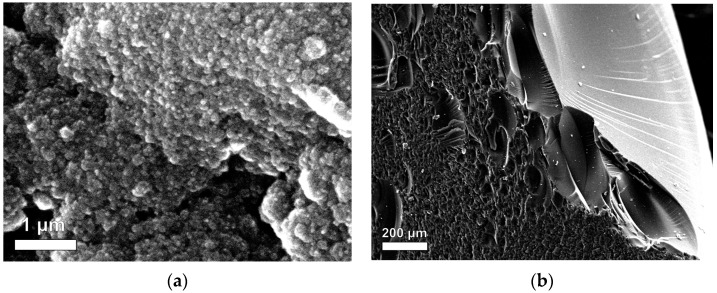
SEM images of RF gels between (**a**) inside and (**b**) outside.

The morphology of RF xerogels (RFX) was observed using scanning electron microscopy (SEM). Figure 2a shows that RFX is composed of a large number of microclusters that are uniformly distributed. These microclusters contain the resorcinol-formaldehyde polymer. The RF gel network is formed with nearly spherical particles, showing similar results to those obtained by [44]. Furthermore, the interconnects between the microclusters were observed to form porous materials. This porosity is likely due to the gelation process used in the synthesis of RFX, which involves the formation of a three-dimensional network of interconnected polymer chains [39,45,46]. Therefore, RFX is a highly porous material with a complex microstructure.

Figure 2a shows the difference between the outside and inside of the RF gel. The microclusters in the outer region appear more compact than those in the inner region. This difference in microstructure is likely due to the contact of the outer region with the glass tube during the gelation process. During gelation, the RF solution is typically poured into a mold or tube and allowed to solidify. The contact of the outer region with the glass surface may have caused the microclusters to pack more tightly together, resulting in a more compact microstructure.

**Figure 3:**
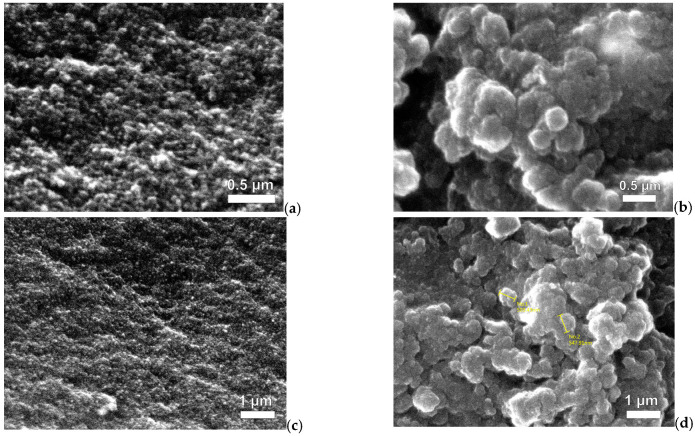
SEM mages of Fe_3_O_4_-monolithic resorcinol-formaldehyde xerogels prepared by (**a**,**c**) direct (MX1) and (**b**,**d**) indirect (MC4) ultrasonication with magnifications.

In this study, the SEM analysis revealed the effect of direct and indirect ultrasonication on the preparation of magnetic xerogels. Figure 3 depicts the morphology of magnetic gels, namely MX1 and MC4, synthesized with the same molar ratios. It can be observed that the morphology of MC4 (Figure 3b,d) characterizes nearly spherical particles that partially overlap, resulting in the formation of large pores. This morphology is likely attributed to the incorporation of magnetite particles into the RF gel during synthesis. Comparing MX1 and MC4 at the same magnification range of 15,000 and 25,000, it is evident that MX1 has smaller particle and pore sizes compared to MC4. Additionally, MX1 shows a more compact RF gel structure than MC4. Both techniques involve delivering energy to the RF solution with magnetite particles through probe sonication. However, the resulting particle sizes and mesoporosity of RF gels differ between the two methods. Indirect sonication generates cavitation in the water bath using high-intensity ultrasound through a water bath, while direct sonication involves the probe causing cavitation during sample processing. It can be explained that the particle size of MX1 decreases after ultrasonication, as observed by [37,47,48].

Energy-dispersive X-ray spectroscopy (EDX) is a technique used to determine the elemental composition of a material. In this study, EDX analysis was used to determine the Fe content, confirming the incorporation of Fe content in the structure of magnetic RF gels. Both magnetic gels demonstrate the physical incorporation of magnetite into the structure of RF gel without participating in the polycondensation reaction of RF, as stated by [47]. MC4 shows a more uniform distribution of magnetite contents in the structure of RF gel than MX1. 

During the synthesis of MX1 with direct sonication, the RF solution was mixed, and the temperature increased dramatically from 45 °C to 79 °C within 5 min, resulting in the formation of a black gel. On the other hand, MC4 was prepared using indirect sonication. The mixed solution of MC1-MC4 allowed the dispersion of magnetite into the RF gel, and the temperature of the solution continuously increased from 33 °C to 85 °C, and finally leading to the formation of a black gel within the water bath for 60 min. 

It can be explained that direct sonication involves the use of a sonication probe directly immersed in the reaction mixture. The probe emits ultrasonic waves that directly interact with the sample, resulting in more localized and precise energy transfer. However, direct sonication generally requires shorter processing times compared to indirect sonication, as the energy efficiently reaches the desired regions, accelerating the required reactions. Consequently, the mixed solution of MX1 with ultrasonication experienced a significant increase in temperature, leading to reduced gelation times [49]. In this study, magnetite was added to the RF solution, and due to its natural behavior, magnetite tends to agglomerate within a short mixing time. Considering the variables involved in the solution, gelation, and curing processes, high temperatures during synthesis lead to porosity shrinkage [49].

**Figure 4:**
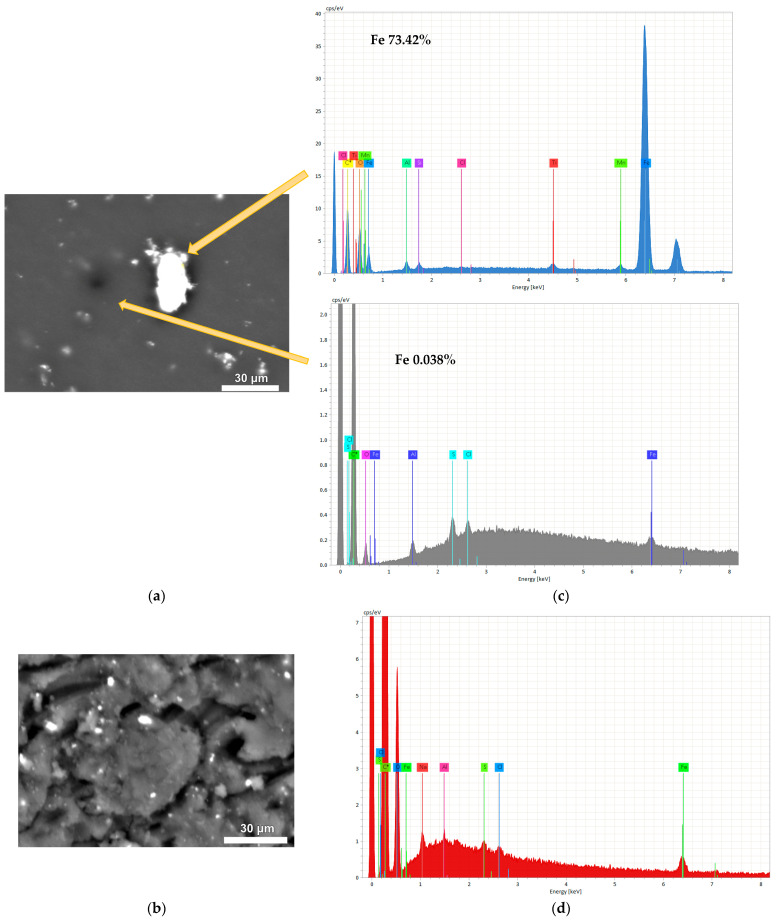
SEM and EDX images of Fe_3_O_4_-monolithic resorcinol-formaldehyde xerogels prepared by (**a**,**c**) direct (MX1) and (**b**,**d**) indirect (MC4) ultrasonication.

After ultrasonication of magnetite into an aqueous RF solution, the particle size of magnetite was decreased, which can be clearly observed with MC4. The EDX analysis revealed that MC4 incorporated 1.19% Fe content (Figure 4d). The M/R ratio used in the synthesis was 0.01, indicating a low concentration of magnetite compared to the RF polymer. This suggests that even with a low M/R ratio, the incorporation of Fe in the RF gel was successful, due to the use of magnetite particles in the synthesis process.

**Figure 5:**
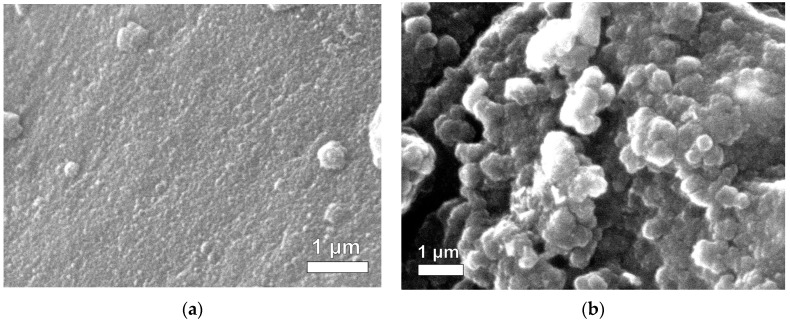
SEM Images of Fe_3_O_4_-monolithic resorcinol-formaldehyde xerogels with R/C ratios of (**a**) 50 (MC1) and (**b**) 200 (MC4).

The morphology of the MCs was studied by SEM as shown in Figure 5. A three-dimensional RF gel network was formed with nearly spherical particles [46,50]. MC1 and MC4 prepared with different Na_2_CO_3_ concentrations, the morphology and pore size distribution can be observed that MC1 with lower R/C molar and high initial pH solution exhibit smaller particles and pore sizes than other materials. pH variations can alter the nucleation and growth of the gel network, leading to changes in the average pore size, pore connectivity, and surface area of the xerogel. Higher pH values can promote the formation of smaller pores, while lower pH values may result in larger pores [50].

The mesoporosity of RF gels increases with an increase in the R/C ratio, as reported in previous studies by [39,45,51]. This indicates that the porosity of RF gels can be controlled by adjusting the R/C ratio in the synthesis process. Mesopores are pores with diameters between 2 and 50 nm and are desirable for various applications such as adsorption.

Table 2 shows the textural properties of magnetic xerogels, the effect of direct, and indirect sonication on the textural properties of materials, specifically MX1 and MC4, respectively. The surface area of the magnetic xerogels for both MX1 and MC4 increased significantly compared to the xerogel. MC4 exhibited a higher surface area of 529.47 m^2^/g, whereas MX1 had a surface area of 472.41 m^2^/g. Additionally, the total pore volume and average pore diameter of MC4 were lower than those of MX1. This can be explained by the fact that MX1, prepared through direct sonication with a shorter sonication time for gelation, resulted in a lower surface area but higher total pore volume and larger average pore size.

The effect of pore structure in magnetic xerogels using the ultrasound-assisted sol-gel method was investigated through N_2_ physisorption analysis. It can be explained that direct sonication promotes the formation of smaller and more uniform pores within the xerogel structure, as the energy can be precisely targeted to specific regions. On the other hand, indirect sonication may result in the generation of larger or more irregularly shaped pores in the xerogel due to less controlled and localized energy transfer. This observation is consistent with the findings in Figure 3 of SEM images and Figure 4 depicting particle distributions, which demonstrate that MX1 has a smaller particle size compared to MC4.

Moreover, Table 2 shows the effect of catalyst contents on the surface area and pore volume of the magnetic xerogels. The results of the RF gels using sodium carbonate as a catalyst show that MC200 had a higher average pore diameter (5.16 nm) than MC100 (4.03 nm), but MC200 had a lower surface area (529.47 m^2^/g) than MC100 (545.09 m^2^/g). However, MC200 (529.47 m^2^/g) exhibited a surface area lower than MC100 (545.09 m^2^/g). These findings are consistent with those of [52], reported that increasing the molar ratios of R/C in gels prepared with Na_2_CO_3_ leads to an increase in average pore width. When lower molar ratios of R/C are used for RF gel preparation, a higher concentration of Na_2_CO_3_ results in the formation of smaller clusters with smaller average-sized pores. Therefore, MC100, with its lower R/C ratios, has a greater number of smaller pore diameters, and a higher surface area, making it more suitable for use in water treatment adsorption.

In this study, the obtained results of the mesoporous nature of magnetic xerogels with varying R/C demonstrated the effect on the surface area and pore volume of the RF polymer in magnetic xerogels. It can be explained that the pH of the RF solution is associated with the quantity of catalyst utilized during the synthesis. When the pH was decreased, both the surface area and pore volume of the RF polymer in xerogels increased. This indicates that lower pH values result in the formation of a greater number of pores and increased surface area within the RF polymer retained in the xerogels [53]. Moreover, it can be explained that the larger carbonate ions have a trigonal planar molecular geometry, which may cause steric hindrance. Consequently, the condensation of the intermediates leads to the generation of larger pores of the samples [54]. The use of a higher amount of catalyst leads to more rapid gelation, resulting in a less uniform structure with fewer and larger pores. Alternatively, the catalyst itself may interfere with the formation of crosslinks within the RF polymer, leading to a less porous structure. Therefore, higher amounts of catalyst used during the synthesis have a similar effect on the surface area and porosity of the resulting material, as observed in the results obtained by [55].

The determination of the isoelectric point (IEP) and point of zero charges (pH_pzc_) of xerogels and magnetic xerogels was carried out by measuring the zeta potential and pH, as shown in Table 2. The IEP and pH_pzc_ of MX1 and MC4 prepared by direct and indirect sonication, respectively, with R/C 200 are in a similar range of values. However, the RF xerogel exhibits lower IEP and pH_pzc_ values compared to the other materials. These findings are similar to the results reported by [56], where organic xerogels demonstrated a pH_pzc_ value of 3.

**Figure 6:**
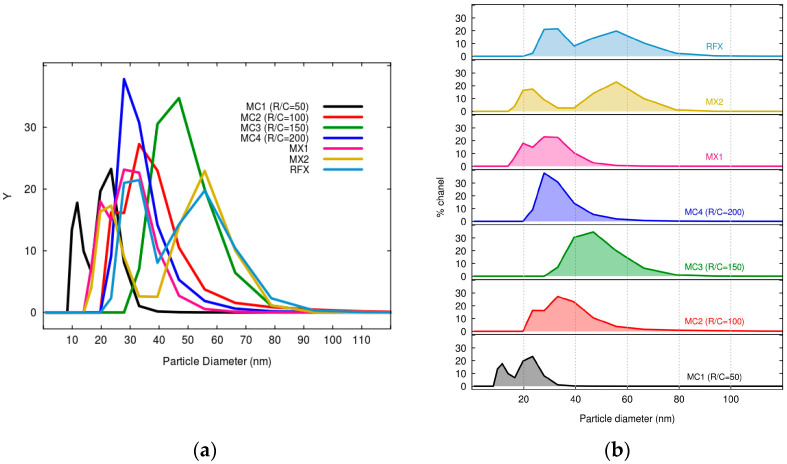
Particle size distribution of RF xerogel and Fe_3_O_4_-monolithic resorcinol-formaldehyde xerogels prepared by the sol-gel method under ultrasonic irradiation and presented in (**a**) grouping and (**b**) separation graphs.

Figure 6 shows the particle distribution of xerogel and magnetic xerogels prepared using the sol-gel method under ultrasonic irradiation. The particle size distribution in the obtained xerogels may vary because of sonication-assisted synthesis and variations in the R/C ratios. RFX exhibits a broader particle size distribution with larger particles compared to MX1 and MC4, which were prepared with the same molar ratios and drying process. RFX, prepared without sonication, showed a larger particle size, which is consistent with the findings of [57].

It can be observed that the average particle diameter of MX1 (28.05 nm) was lower than that of MC4 (32.65 nm), which is similar to the results obtained from SEM analysis. The use of direct sonication in the preparation of MX1 resulted in a narrower particle distribution due to localized energy transfer, leading to more consistent particle sizes in the obtained xerogel. On the other hand, MC4 exhibited a wider range of particle sizes due to less precise control of sonication energy distribution. Therefore, the direct method of sonication generally leads to a lower particle size distribution compared to the indirect method, due to the more localized and intense energy transfer that promotes effective fragmentation and reduction in particle size. Similar findings of the study of [58].

The initial pH of the solution is a factor influencing the polymerization of xerogels, especially when varying the molar ratio of the catalyst. The pH values of the RF solutions for MC1, MC2, MC3, and MC4 were 7.26, 7.05, 6.92, and 6.82, respectively, within the similar range of the study of [52]. It can be observed that higher catalyst concentrations with lower R/C molar ratios result in smaller particles and pore sizes, as reported by [53].

The pH of the precursor solution plays a crucial role in determining the final structure of the obtained xerogel. It affects the kinetics of polymerization and crosslinking reactions, as well as the condensation and gelation processes. The mechanism of polymerization in RF gels involves two steps: the addition reaction to form hydroxymethyl derivatives of resorcinol and the condensation of these derivatives to form methylene or methylene ether bridged compounds [49]. In a high pH solution, the first addition reaction is favored. This leads to a higher rate of polymerization and crosslinking, resulting in a more extensively crosslinked network structure and a relatively quick process. This process often yields small nodules and narrow mesopores. Gelation kinetics, which refers to the rate of transition from a liquid precursor solution to a gel network, is strongly influenced by pH. Higher pH values generally promote faster gelation, while lower pH values slow down the process. The gelation kinetics can significantly impact the overall pore structure and porosity of the xerogel. When the condensation reaction occurs in the presence of small particles resulting from the high pH conditions, it produces materials with smaller pores, leading to a higher density or more compact RF gel structure [59]. On the other hand, lower pH values may result in a less densely crosslinked structure.

**Figure 7:**
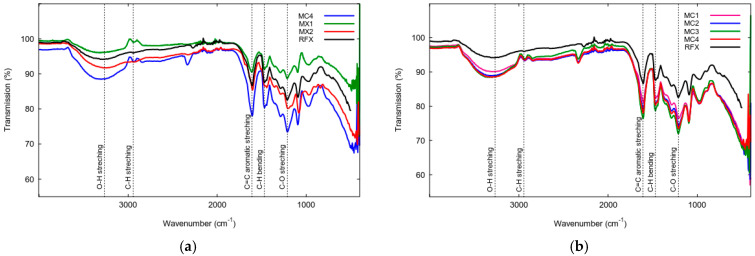
FTIR spectra of RF xerogel and Fe_3_O_4_-monolithic resorcinol-formaldehyde xerogels prepared by (**a**) direct and indirect sonication ultrasonication-assisted and (**b**) varying of R/C molar ratios.

Figure 7a depicts the FTIR spectra of RFX and magnetic gels prepared using ultrasonication with direct and indirect techniques, covering a wavelength range of 4000–400 cm^−1^. The characteristic FTIR bands of RFX, MX, and MC are similar. However, MC4, MX1, and MX2 exhibit an FTIR band at 478 cm^−1^ attributed to Fe-O stretching vibration [60,61]. The profiles of RFX, MC4, and MXs show the presence of six absorption bands: (i) O-H stretching at 3300 cm^−1^, (ii) C-H stretching at 2900 cm^−1^, (iii) C = C stretching in the aromatic ring at 1600 cm^−1^, (iv) C-H bending vibration at 1400 cm^−1^, (v) C-O stretching at 1200 cm^−1^, and (vi) methylene ether C-O-C linkage stretching between two resorcinol molecules at 1000 cm^−1^ [62]. The FTIR spectra of RF gel and MC1-MC4 can be observed in Figure 7b, and all of them exhibit bands that are correlated with the bands described above.

Regarding the characterization of MCs and MXs, it can be observed that the preparation of monolithic resorcinol-formaldehyde xerogels involved different methods of sonication, utilizing low and high intensity, respectively. However, the results of their XRD and FTIR analyses show significant similarities. This is in contrast to the study conducted by [48], where the preparation of ZnO nanoparticles using direct and indirect sonication had an impact on the crystalline structure (XRD analysis) and resulted in different IR spectra of the samples. Due to the probable growth mechanisms of ZnO nanoparticles, various crystallization mechanisms were proposed. However, in the case of xerogels, ultrasonic irradiation aids in promoting aging and hydrophobization reactions. Additionally, [37] discovered that the preparation of silica xerogels can be accomplished in less than 1/5 of the time required by conventional methods.

#### 2.1.2. Performance of Adsorption of Arsenic Using MCs and MXs

**Figure 8:**
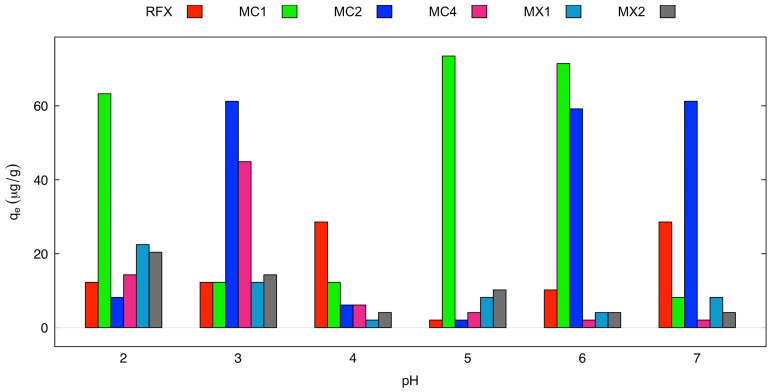
Effect of pH on the adsorption of As(V) using Fe_3_O_4_-monolithic resorcinol-formaldehyde xerogels (Condition: initial concentration 100 µg/L, dose 1 g/L, 6 h, and temperature 25 °C).

In the batch adsorption experiment of As(V) using MCs and MXs, the effect of pH in the range of 2 to 7 was used to evaluate their adsorption capacities, as shown in Figure 8. MC1 and MC2 demonstrated high adsorption capacities, q_e_ were more in the range of 63.26–73.47 µg/g and 59.18–61.22 µg/g, respectively, than other materials. MX1 and MX2 showed higher adsorption capacity in the acidic solution. Due to the pH_pzc_ being the zero net charge on the surface of the adsorbent, the adsorbent surfaces are charged positively or negatively, depending on whether the pH of the solution is lower or higher than the pH_pzc_ values, respectively [55]. The analysis result of pH_pzc_ of MX1 was 4.54, meaning that MX1 adsorbed As(V) at pH values lower than this value. The same can be described for the adsorption of MC1 and MC2, whose pH_pzc_ values were 6.63 and 6.12, respectively.

### 2.2. Fe_3_O_4_-Monolithic Resorcinol-Formaldehyde Xerogels with Direct and Indirect Sonications: Effects of Power Output of Ultrasonic Processor, Varying the Molar Ratios of M/R and R/W

Table 3 presents the molar ratios used in the synthesis of five magnetic xerogels, specifically MX3-MX7. These xerogels were prepared with molar ratios of M/R of 0.03, 0.05, 0.1, 0.15, and 0.2, respectively. The xerogels were synthesized using direct ultrasonic-assisted synthesis with an ultrasonic VCX130 operating at 130 watts and a 1/4” diameter probe. The M/R ratios increased with the increasing Fe contents, as determined by chemical composition analysis using ICP-Optical Emission Spectroscopy. However, MX6 and MX7 exhibited similar Fe content values. In this case, it can be explained that the high quantity of magnetite may not have fully incorporated into the gel matrix and some of it may have washed out during the solvent exchange, as evidenced by the observation of a brown solution after changing the acetone solution.

MX8-MX11 were synthesized using indirect sonication via the Q700 sonicator, which has a power output of 700 watts and a 1/2” diameter probe. The molar ratios used in the synthesis, along with the corresponding Fe content, are shown in Table 4.

At the same molar ratios of M/R at 0.15 for direct (MX7) and indirect (MX11) sonication, MX11 demonstrated a higher Fe content than MX7. The theoretical calculations of Fe content for MX8, MX9, MX10, and MX11 are 4.27%, 6.85%, 12.52%, and 17.29%, respectively. These values are similar to the results obtained from ICP-OES analysis. This can be explained that increasing the power output to 700 watts makes the system more homogenous. 

#### 2.2.1. Characterization of Fe_3_O_4_-Monolithic Resorcinol-Formaldehyde Xerogels MX3-MX7 and MX8-MX11

**Figure 9:**
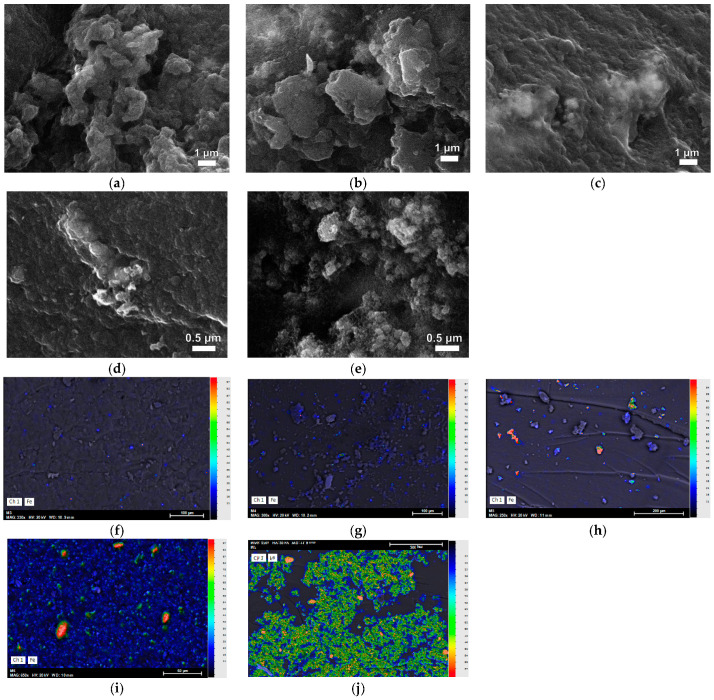
SEM images and EDX mapping analysis of magnetic xerogels prepared with R/C = 200, R/W = 0.04, and varying M/R of MX3 = 0.03 (**a,f**), MX4 = 0.05 (**b,g**), MX5 = 0.1 (**c,h**), MX6 = 0.15 (**d,i**), and MX7 = 0.2 (**e,j**), respectively.

SEM images and mapping analysis of MX3-MX7 are shown in Figure 9, with an increasing amount of magnetite through direct sonification. Figure 9a–e shows the SEM images of the surface morphology of magnetic xerogels composed of large numbers of microclusters with a three-dimensional network. However, some parts of them are agglomerated, and some bright particles can be observed. The elemental distribution of these particles can be confirmed with the corresponding EDX spectra, which demonstrate the existence of iron (Fe), oxygen (O), carbon (C), aluminum (Al), and sodium (Na).

Elemental mapping was analyzed to observe the distribution of synthesized magnetite on RF matrix gels, as shown in Figure 9f–j. The individual EDX mapping of Fe element distributions: blue = low, green = medium, and red = high. Magnetite particles were observed in a blue color and were evenly distributed on the RF surface, with a higher quantity corresponding to the increasing M/R ratios. Similar results were obtained from Table 3. The results of the mapping analysis show that the incorporation of Fe into the structure of the samples is homogeneously distributed, similar to the results obtained from activated carbon xerogels doped with iron (II) phthalocyanine by ultrasonication [63]. However, some of them had some accumulation of Fe due to the increase of high concentration of magnetite in the RF solution. It can be observed in Figure 9h,i, where EDX mappings for Fe display a red color in several regions, indicating a high concentration of Fe within the RF gels. The agglomeration of the microclusters and the presence of bright particles on the surface of the MX5-MX7 xerogels suggest that the synthesis process could be improved. Further studies are needed to optimize the synthesis conditions in order to produce magnetic xerogels with improved properties.

**Figure 10:**
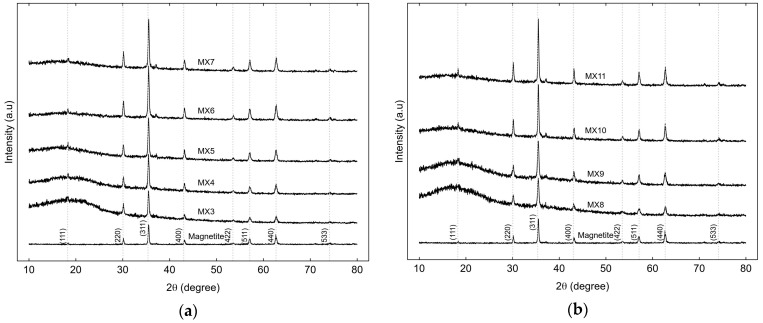
XRD patterns of magnetic xerogels prepared by (**a**) direct (MX3-MX7) and (**b**) indirect (MX8-MX11) ultrasonic with varying M/R ratios of 0.03–0.2.

The XRD patterns of MX3-MX7, prepared by direct sonication, and MX8-MX11, prepared by indirect sonication, are shown in Figure 10a,b, respectively. Both sets of samples were synthesized with different molar ratios and utilized different ultrasonic processors. However, both sets varied the M/R ratios from 0.03 to 0.2 for MX3-MX7 and from 0.03 to 0.15 for MX8-MX11. Consequently, the XRD analysis of the magnetic xerogels demonstrated the presence of magnetite, in accordance with the JCPDS card assignments, as described in Figure 1. The diffraction peaks at d311 (2 θ = 35.68°) appeared high and sharp for all materials, indicating their magnetic properties [42], the intensity of the iron phase peaks increased with higher M/R ratios in the synthesis. These results are particularly relevant for the analysis of the chemical composition, as presented in Table 3 and Table 4.

**Figure 11:**
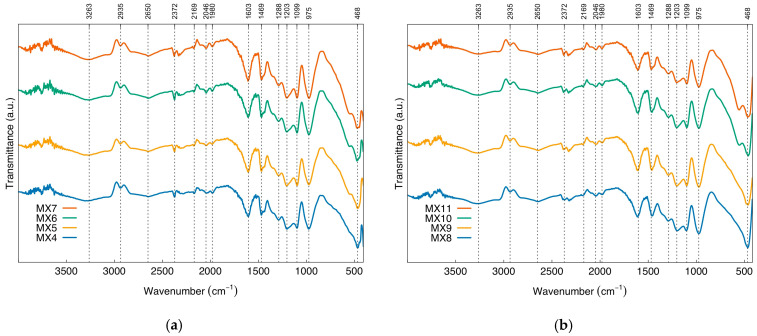
FTIR Spectra of magnetic xerogels prepared by (**a**) direct (MX4-MX7) and (**b**) indirect sonication (MX8-MX11).

The FTIR spectra of monolithic resorcinol-formaldehyde xerogels prepared by direct sonication, with varying M/R molar ratios of 0.05, 0.1, 0.15, and 0.2 (referred to as MX4, MX5, MX6, and MX7, respectively) are similar, as shown in Figure 11a. Similarly, Figure 11b presents FTIR spectra of MX8-MX11, prepared by indirect sonication, which exhibit similarities. The resulting FTIR spectrum displays peaks corresponding to different vibrational modes of the molecules in the sample, as discussed in detail in Figure 7. Both groups of materials exhibit an FTIR band at 468 cm^−1^, attributed to Fe-O stretching vibration [60,61]. Therefore, the use of different sonication methods and power outputs of the ultrasonic processor has no effect on the functional groups and chemical compounds present in the samples of monolithic resorcinol-formaldehyde xerogel, based on the absorption of infrared radiation with wavelength ranges of 4000–400 cm^−1^. 

#### 2.2.2. Performance of Adsorption of Arsenic Using MX4-MX7 and MX8-MX11

**Figure 12:**
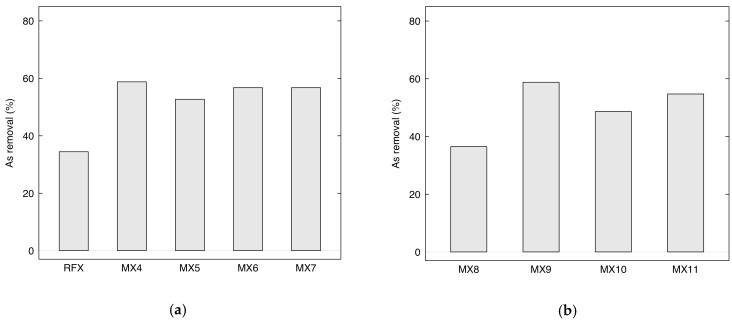
As(V) removal using Fe_3_O_4_-monolithic resorcinol-formaldehyde xerogels prepared by (**a**) direct sonication with low power output and (**b**) indirect sonication with high power output. (Conditions: initial concentration 200 µg/L, pH of 3, dose 2 g/L, 6 h, and temperature 25 °C).

Figure 12a presents the removal efficiency of As(V) using MX4-MX7 prepared by direct sonication via a sonicator with 130 watts of power. The removal efficiency of MX4-MX7 was higher than RFX, which was prepared without using magnetite. In particular, MX4 with a lower loading of Fe_3_O_4_ (M/R = 0.03) gave the highest arsenic removal of 58.78%. Meanwhile, arsenic removals were lower with MX5, then increased and remained constant for MX6 and MX7. This can be explained by the capacity of the sonicator. With a low power output sonication and small diameter tip, it was possible to homogenize the solution well with a low quantity of magnetite. However, with increasing magnetite loading into the RF solution with M/R of 0.05, 0.07, and 0.15, the As removal results were similar. This can be confirmed with SEM/EDX analysis (Figure 9), which showed that magnetite was more homogeneously distributed in MX4 than in the other materials.

Figure 12b shows the arsenic removal using MX8-MX11 prepared by indirect ultrasonic-assisted synthesis with 700 watts. The removal of MX8 (M/R = 0.03) and MX9 (M/R = 0.05) increased dramatically from 36.49% to 58.78%. With the increasing of M/R to 0.1 and 0.15, their arsenic removal of MX10 and MX11 remained constant, which demonstrated the same behavior as MX4-MX7.

Additionally, the effect of the molar ratio of R/W and M/R on the total solids content of the materials is shown in Table 3 and Table 4. The solid content increases with increasing magnetite loading. At the same molar ratios of M/R, the total solids content also increases with increasing R/W. Moreover, low solids contents result in fragile structures, and very high solids contents result in increased densification of the material that lowers porosity. Therefore, the optimum solids content of the xerogel is 20 *w*/*v*% [45].

### 2.3. Fe_3_O_4_-Monolithic Resorcinol-Formaldehyde Xerogels and Carbon Xerogels by Indirect Sonication

#### 2.3.1. Characterization of Fe_3_O_4_-Monolithic Resorcinol-Formaldehyde Carbon Xerogels

**Figure 13:**
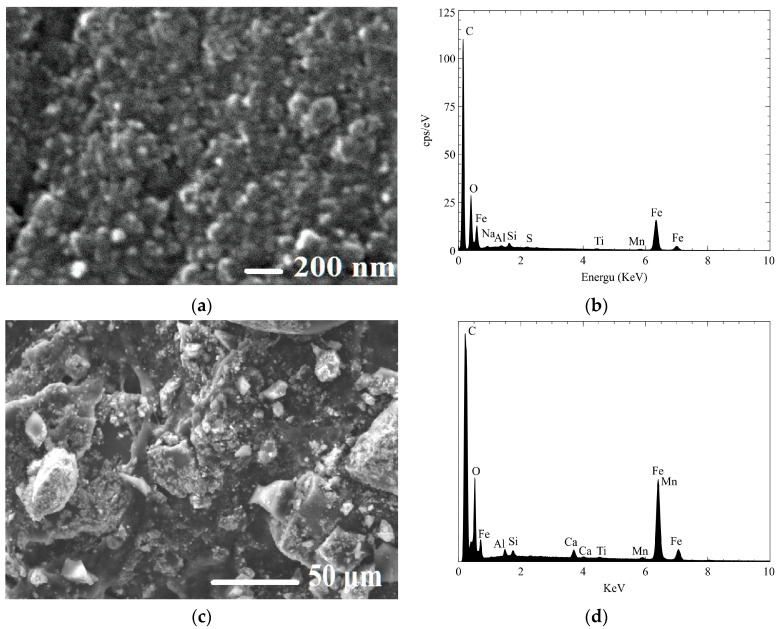
SEM and EDAX images of (**a**,**b**) Fe_3_O_4_-Monolithic resorcinol-formaldehyde xerogels (MXRF), and (**c**,**d**) Fe_3_O_4_-Monolithic resorcinol-formaldehyde carbon xerogels (MXRF600).

Some parts of the RF surface of Fe_3_O_4_-Monolithic resorcinol-formaldehyde xerogels (MXRF) were agglomerated due to the formation of magnetite, as shown in Figure 13a. The presence of Fe in the RF gels was determined to be 14.83 w% by AAS, compared to 24.67% of Fe as quantified by EDX in the solid sample. It can be observed that the morphology and EDAX analysis did not change significantly after the adsorption process (Figure 13b).

**Figure 14:**
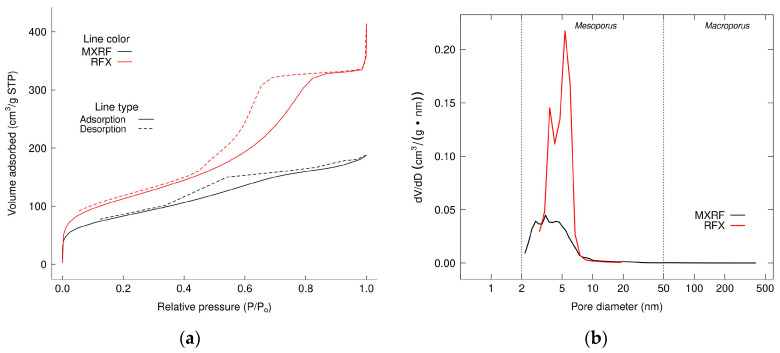
(**a**) N_2_ adsorption isotherms and (**b**) pore size distributions of xerogel (RFX) and Fe_3_O_4_-monolithic resorcinol-formaldehyde xerogels (MXRF).

Figure 14a shows N_2_ adsorption–desorption isotherms at 77 K, and Figure 4b illustrates the pore size distributions of XRF and MXRF. The analysis results of BET surface area, total pore volume, and average pore size of xerogel adsorbent were 399.19 m^2^/g, 0.517 cm^3^/g, and 5.228 nm, respectively. When magnetite composites were added to xerogels, the porous properties of MXRF for BET surface area, total pore volume, and average pore diameter were 292 m^2^/g, 0.279 cm^3^/g, and 3.81 nm, respectively.

Figure 14a shows that the adsorption isotherms of RFX and MXRF adsorbents at a constant temperature of 77 K with N_2_ as the adsorptive exhibit a linear relationship between relative pressure and amount adsorbed. RFX and MXRF exhibited type IV adsorption isotherms with H2 and H4 hysteresis loops, respectively. This implies that RFX contained typical mesoporous materials and MXRF contained micro- and mesoporous adsorbents, similar to the results of the pore size distributions.

Figure 14b shows that the main pore diameter sizes of RXF and MXRF are in the range of 2–50 nm, which is defined as mesoporous material. The pore size distribution of MXRF reveals that the average pore diameter was 3.81 nm, which is similar to the results of the narrow centering of PSD of Fe, Co, and Ni doped carbon xerogels [64]. This indicates that the doping with transition metals, such as magnetite, into the xerogels has a similar effect to the composite of magnetite, which affects the reduction of surface areas and total pore volume of the material and makes alterations to their textural properties [65,66]. Similar results were found from SEM analysis, which showed increased agglomeration of particles in RF gels.

**Figure 15:**
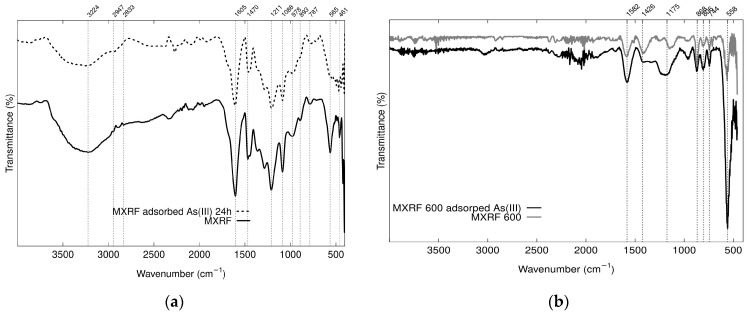
FTIR analysis before and after adsorption of As(III) of (**a**) magnetic xerogels of resorcinol formaldehyde (MXRF) and (**b**) magnetic carbon xerogels of resorcinol formaldehyde (MXRF600).

As shown in Figure 15, FTIR analysis of MXRF before and after adsorption of As(III) was obtained using attenuated total reflection (ATR) technique. Absorption peaks at 558 cm^−1^ are characteristic peaks of Fe-O-Fe, which are indicative of magnetite, confirming the presence of Fe_3_O_4_ on the MXRF adsorbent [67]. The bending vibration of the hydroxyl groups (Fe–OH) confirmed the formation of iron oxide in xerogels [68] and O–H groups on the gel surface. These groups are possible to facilitate the adsorption of arsenic by iron oxides composites in the matrices of RF magnetic xerogels [28].

**Figure 16:**
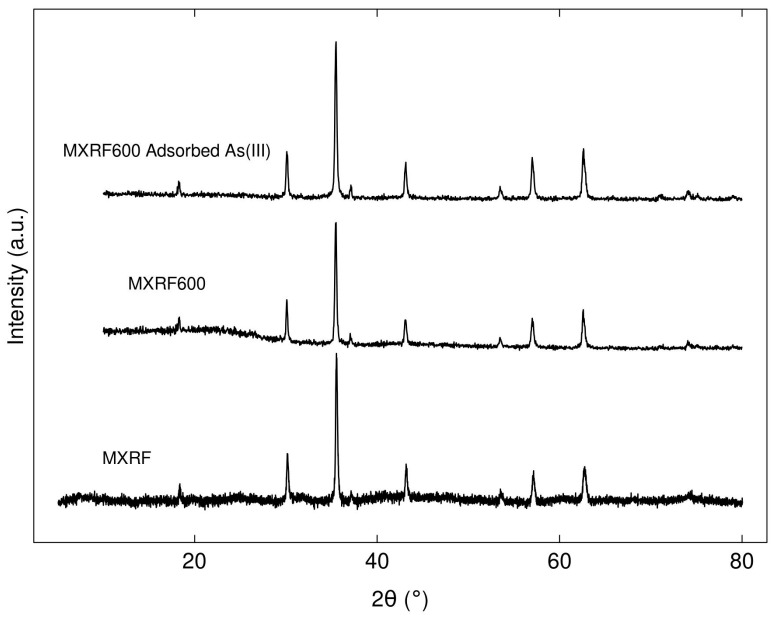
XRD diffractogram of Fe_3_O_4_-monolithic resorcinol-formaldehyde xerogels (MXRF) and carbon xerogel (MXRF600) before and after adsorption of As(III).

XRD patterns of MXRF and MXRF600 before and after adsorption (Figure 16) clearly demonstrated that they had high intensity peaks that contained a crystalline phase and corresponded to Fe_3_O_4_ with the Joint Committee on Powder Diffraction Standards (JCPDS) card No. 19-0629. Therefore, the chemical and structural properties of MXRF and MXRF600 did not change significantly following the carbonization and adsorption process.

#### 2.3.2. Adsorption of Low and High Concentration of As(III) and As(V) with MXRF and MXRF600

In the adsorption process, contact time is one parameter that is a time-dependent process. Adsorption kinetic studies are important in water treatment. These studies can describe the mechanism of the adsorption process and provide kinetic adsorption constants and valuable information. The experimental data were analyzed with four kinetic models: pseudo first-order, pseudo second-order, Elovich, and Power function. 

**Figure 17:**
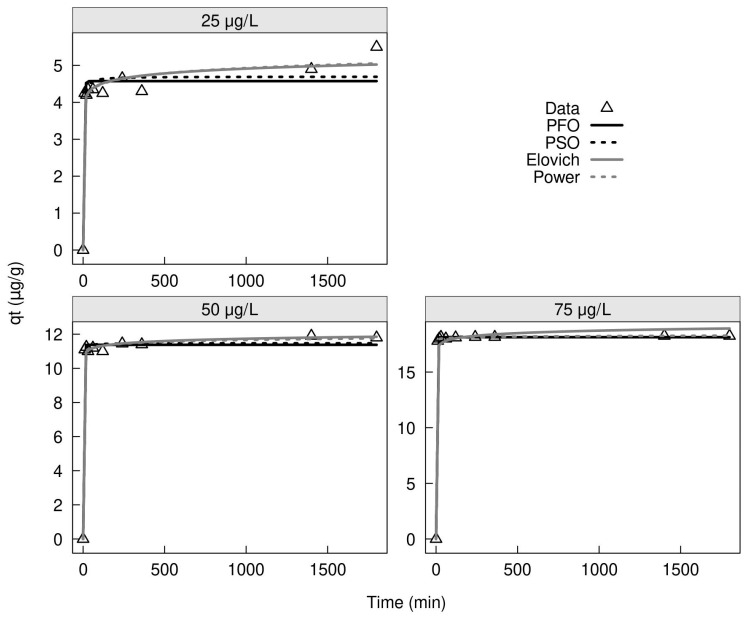
Adsorption kinetics using MXRF with 0.025, 0.05, and 0.075 mg/L of As(III) concentrations, pH of 3, and dosage of 2 g/L.

The effect of contact time on the adsorption process was varied from 10 to 1440 min with different ranges of initial concentration for the low range of As(III) concentrations (25, 50, and 75 µg/L) and high range of concentration for As(III) and As(V) were 514 µg/L and 1034 µg/L, respectively. The adsorption kinetic of As(III) on MXRF is shown in Figure 17. The removal efficiency for As(III) concentration of 75 µg/L increased faster in 10 min and remained constant until 240 min at 97.33%. 

Kinetic parameters and correlation coefficients for As(III) and As(V) adsorption by using MXRF600 were obtained by nonlinear regression as presented in Table 5, including residual root mean square error (RMSE). The condition of As(III) and As(V) adsorption kinetics were pH of 3, dosage of 2 g/L, and initial concentration of As(III) and As(V) solution of 0.514 mg/L and 1.034 mg/L, respectively. The adsorption kinetic models that presented the best fit in the As(III) and As(V) adsorption process were the Power equation and Elovich chemisorption model.

It can be observed that MXRF600 demonstrated greater adsorption of As(III) and As(V) than MXRF, implying a higher adsorption capacity. The final step of preparing MXRF600 was to produce a carbon xerogel with a carbonization process for removing the rest of the oxygen and hydrogen groups and improving a thermally stable nanostructure [49]. With the use of high temperature under an inert atmosphere, MXRF and MXRF600 demonstrated modifications in their chemical composition and texture properties, which can be identified with the analysis of XRD, FTIR, N_2_ physisorption, and SEM/EDAX, as discussed above.

In this study, the experimental data were analyzed with nonlinear equations using the Langmuir and Freundlich isotherm models to describe the adsorption of As(III) and As(V) on MXRF600. The Langmuir isotherm model assumes that a monomolecular layer of adsorbate molecules is formed on the adsorbent surface, with each molecule having the same adsorption energy. The Freundlich isotherm model describes the heterogeneity of the surface and the distribution of adsorption energies.

The conditions for the isotherm adsorption were as follows: adsorbent dose of 2 g/L, initial solution pH of 3.0, and contact time of 24 h. The initial concentrations of the As(III) and As(V) solutions were in the range of 0.05–1.27 mg/L and 0.12–3.0 mg/L, respectively. The Langmuir and Freundlich model parameters and regression coefficients are shown in Table 6. The experimental data for the adsorption of As(III) and As(V) on magnetic carbon xerogel monoliths were fitted to the Langmuir models, and the maximum monolayer adsorption capacity (q_max_) of As(III) and As(V) were 694.3 and 1720.3 µg/g, respectively, with R^2^ values (RSME) of As(III) and As(V) were 0.897 (3.865), and 0.901(9.220), respectively. 

## 3. Conclusions

The ultrasonic-assisted synthesis of Fe_3_O_4_-monolithic resorcinol-formaldehyde xerogels using direct and indirect sonication methods as an easier recovery of adsorbent was shown to reduce the gelation time and improve the textural properties of the final product. The optimal mixing time for magnetite dispersion in an RF aqueous solution was determined to be 5 min using direct sonication and 60 min using indirect sonication, as confirmed by SEM/EDX analysis. This study investigated the effect of different molar ratios of R/C, M/R, R/W, and thermal treatment on RF xerogel. The results show MXRF600 was synthesized by indirect sonication with R/F = 0.5, R/C = 100, R/W = 0.05, and M/R = 0.15 and enhanced adsorption capacity for As(III) and As(V) from groundwater due to the influence of sonication assistance and the carbonization process. However, the optimization of the process parameters for the adsorption of magnetic carbon xerogels should be studied to find out the optimum condition and improve their performance in removing contaminants from the environment. The desorption process, regeneration efficiency, and the lifecycle assessment of magnetic carbon xerogels are suggested for future research.

## 4. Materials and Methods

### 4.1. Reagents and Materials

Reagents required to perform the synthesis of Fe_3_O_4_ nanoparticles were prepared in duplicate, including ferric chloride hexahydrate (FeCl_3_·6H_2_O, 98.9%, Fermont), ferrous sulfate heptahydrate (FeSO_4_.7H_2_O, 99%, Meyer), and sodium hydroxide (NaOH, 97%, Meyer. Nitrogen gas was purchased from Infra (Morelos, Mexico). Resorcinol (C_6_H_4_(OH)_2_, 98%, Chemistry Meyer), sodium carbonate (Na_2_CO_3_, J.T. Baker, 100%), formaldehyde (HCHO, 37% methanol stabilized Solution, J.T. Baker), acetone ((CH_3_)_2_CO, 99.5%, J.T. Baker,), and magnetite Fe_3_O_4_ (Lanxess, Bayferrox) were used for synthesis of magnetic xerogels. All the solutions used in the synthesis and adsorption experiment were made using ultrapure Type I water from the water purification system (WaterproBT, Labconco, Kansas City, MO, USA).

### 4.2. Synthesis of Adsorbent Materials

The gels were synthesized by polymerizing resorcinol (R, C_6_H_6_O_2_) and formaldehyde (F, CH_2_O) in water (W), using sodium carbonate (C, Na_2_CO_3_) as a catalyst, following the procedure described by [69]. The synthesis utilized molar proportions of R/C = 200, R/F = 0.5, and R/W = 0.06 [70,71]. While keeping other factors constant, the effect of loading of Fe (via direct and indirect sonication), Fe content (M/R = 0.03–0.2), water (R/W = 0.04–0.06), and catalyst (R/C = 50–200) ratios were varied in the realization of the monoliths. Then, they were evaluated for their impact on the physicochemical properties of the resulting materials, as well as their ability to remove As(III) and As(V). Iron oxides (M) used in this study were magnetite obtained from Lanxess, García, Nuevo León, Mexico.

The procedure for synthesizing the gels involved placing half of the deionized water and the mass of R in a 100 mL beaker, which was then vigorously shaken to homogenize the solution. The F solution was added, followed by the addition of C, and the mixture was stirred magnetically until homogeneous. pH of RF solution was controlled between 5.5–6.0 to obtain high surface areas of resulting materials [52]. The resulting solution was then placed in Pyrex^®^ glass tubes, which were sealed with a stopper to prevent evaporation. The temperature of the RF solution during reaction of an ultrasonic processor was controlled to be in the range of 80 to 85 °C. To evaluate the optimal mixing time and the dispersion of magnetite in the RF aqueous solution, three types of ultrasonic devices were applied. First, a digital ultrasonic device (UP400St; Hielscher, Teltow, Germany) with an output of 400 watts, a frequency of 24 kHz, and a 1-inch diameter probe was used in the synthesis of RFX, MC1-MC4, MX1-MX2, and MXRF. The device was equipped with automatic frequency tuning and adjusting an amplitude ranging from 80% to 100%. An ultrasonic processor (VCX 130; Sonics & Materials, Inc., Newton, CT, USA) with a power output of 130 watts, a frequency of 20 kHz, and a ¼-inch diameter tip was applied in the synthesis of MX3-MX7. A sonicator (Q700; Qsonica L.L.C, Newtown, CT, USA) with a power rating of 700 watts, a frequency of 20 kHz, and a 1/2-inch diameter probe was used in the synthesis of MX8-MX11. All ultrasonic processors were used for homogenization, dispersal, and deagglomeration of magnetite particles in the RF aqueous solution, using both direct and indirect sonication methods before the gelation process.

#### 4.2.1. Monolithic Resorcinol-Formaldehyde Xerogels Effect of Loading of Magnetite with Direct and Indirect Sonication, and Modification of Catalyst

The study investigated the optimal mixing time and dispersion of magnetite in RF aqueous solution, using both direct and indirect ultrasonication methods prior to the gelation process. Magnetic xerogel monoliths (MCs) were prepared by indirect sonication with molar ratios of R/F = 0.5, R/W = 0.06, and M/R = 0.01, and varying proportions of resorcinol and catalyst. MC1, MC2, MC3, and MC4 were identified based on R/C ratios of 50, 100, 150, and 200, respectively. The homogenization process was carried out using ultrasonic-assisted synthesis, with digital ultrasonic equipment (UP400St; Hielscher, Teltow, Germany), starting at room temperature. After 5 min of sonication, the temperature reached 85 °C. Magnetite (M) was added into the homogeneous RF aqueous solution and subjected to indirect sonication for 60 min to disperse the magnetite particles before the gelation process. Additionally, the variable factors studied in this work include loading of magnetite with direct and indirect ultrasonication. Therefore, MX1 was prepared using the same method as MC4 but with direct sonication to compare their properties and adsorption capacity of arsenic in aqueous solution. Afterward, the materials were placed in the oven at 80 °C for 5 days. In the case of MX2, the gelation and curing process was changed to be left at room temperature for 5 days.

#### 4.2.2. Monolithic Resorcinol-Formaldehyde Xerogels with Direct and Indirect Sonication: Effects of Power Output of Ultrasonic Processor, Varying the Molar Ratios of M/R and R/W

In this study, monoliths of magnetic xerogels (MXs) were prepared by the sol-gel polymerization of resorcinol with formaldehyde, using an alkaline catalyst and direct sonication of magnetite to incorporate them into the xerogels. Different proportions of iron oxides were modified to achieve the maximum adsorption capacity. Initially, batches of magnetic xerogel monoliths (MX3-MX7) were prepared by varying the M/R ratio from 0.03 to 0.2. The molar ratios of R/F = 0.5, R/C = 200, and R/W = 0.04 were maintained for a small portion of the batches. The preparation process involved the use of an ultrasonic processor VCX 130 with a power output of 130 watts and a frequency of 20 kHz.

#### 4.2.3. Monolithic Resorcinol-Formaldehyde Carbon Xerogels by Indirect Sonication

The monolithic resorcinol-formaldehyde xerogels (MXRF) were synthesized in a larger batch using UP400St equipment with the relations of molar ratio of R/F = 0.5, R/C = 100, R/W = 0.05, and M/R = 0.15. Then, the gels were cured in a conventional oven for three days at 80 °C. The gels were taken off the glass tubes and allowed to cool to room temperature. After that, the gels were cut using a diamond disk into pellet forms of 5 mm in diameter. The materials were then exchanged with acetone, sealed in a jar with the lid tightly closed, and wrapped with paraffin film. The jar was placed in a shaking water bath (BS-11; Lab Companion, Daejeon, Republic of Korea) at 150 rpm for two days, with fresh acetone being added daily. Subsequently, the gels were dried for three days in a conventional oven at 80 °C. 

MXRF were then pyrolyzed using a tube furnace (STF55346C-1; Lindberg/Blue M, Asheville, NC, USA) with the following conditions: temperature of 600 °C, heating ramp of 3 °C/min, time of 6 h, and nitrogen flow of 100 mL/min. The resulting product was monolithic resorcinol-formaldehyde carbon xerogels, which were labelled as MXRF600.

### 4.3. Characterization through Analytical Techniques

To assess the physicochemical characteristics of the synthesized materials, the following techniques were employed:

X-ray diffraction (XRD) analysis was used to identify the main constituents and mineralogical phases of the synthesized materials. The analysis was performed using an X-ray diffractometer on MCs, MXs, and MX3-MX11 samples (XPert PW3040; Philips, Almelo, The Netherlands), and on MXRF and MXRF600 (D8 ADVANCE; Bruker, Karisruhe, Germany). Sample preparation involved sieving the sample through a 200-mesh sieve, resulting in an average particle size of 74 µm. A high-temperature chamber attached to the X-ray diffractometer was used to measure diffraction patterns up to 900 °C. Cu(Kα) radiation was applied in a 2θ range from 10° to 80°.

Fourier transform infrared spectroscopy (FTIR) was employed to investigate the surface functional groups of the adsorbents before and after arsenic adsorption, in order to understand the mechanism of ion adsorption. FTIR analysis was conducted using a Shimadzu IRAffinity-1S instrument (Shimadzu Corp., Kyoto, Japan) on dry powder samples. Infrared spectra were measured by connecting to the attenuated total reflection (ATR) contained in the disk of crystal (type IIIa monocrystalline diamond). Before the analysis, the samples were sieved through a standard test sieve No. 142 to obtain a uniform particle size of 106 μm. Subsequently, the powder samples were dried in an oven at 60 °C for 15 h under dry air to avoid interference from water vapor adsorption in the infrared region, which could affect the analysis result. After installing the ATR with infrared spectroscopy, the solid samples were directly added to the crystal plate and pressed for surface analysis. All spectra were recorded between the wavenumbers of 400–4000 cm^−1^, with 45 scans per sample. 

The surface morphology, pore structure, and element analysis of the magnetic xerogels were analyzed using a scanning electron microscope (SEM). MCs, MXs, MXRF, and MXRF600 were analyzed using a field emission scanning electron microscope (FE-SEM) (7800F Prime; JEOL, Tokyo, Japan) after gold coating. MX3-MX11 were analyzed using a scanning electron microscope (SEM) (JSM-IT300; JEOL, Tokyo, Japan). The samples were coated with graphite before the analysis. The acceleration voltages used were between 5 and 20 kV. The textural properties of magnetic xerogels, and magnetic carbon xerogels were characterized by physical adsorption of N_2_ at 77 K, using physisorption apparatus (ASAP 2020; Micromeritics, Norcross, GA, USA and NOVA touch 2LX; Quantachrome Instruments, Boynton Beach, FL, USA). The samples were dried at 110 °C for 15 h prior to N_2_ physisorption analysis.

Particle Size Distribution (PSD) was determined using Dynamic Light Scattering Analyzers (PMX 500; Microtrac, Meerbuch, Germany), and the data report was generated by FLEX software version 11.1.0.1 

The determination of the point of zero charge (pH_pzc_) and the isoelectric point (IEP) was conducted following the methods described by [72]. The pH solutions were prepared by adjusting deionized water to pH values of 2, 4, 6, 8, 10, and 12 using 0.1 M HCl or 0.1 M NaOH solutions. The pH was measured using a multi-parameter device (Orion Star A211; Thermo Scientific, Beverly, MA, USA). A zeta potential analyzer (PMX 500; Microtrac, Meerbuch, Germany) was employed to measure the zeta potential, with pH variations ranging from 2 to 11 for the determination of the isoelectric point (IEP).

The amount of Fe in the magnetic xerogel monoliths was determined by Inductively Coupled Plasma (ICP) Optical Emission Spectrometer (OES) (Optima 8300; Perkin Elmer, Shelton, CT, USA).

### 4.4. Batch Adsorption Experiment

Groundwater used in experimental study was obtained from a well approximately 70 m deep located at Jiutepec, Morelos Mexico. Physical and chemical characteristics of groundwater sample used in this study were analyzed. pH (7.6), total dissolved solids (TDS, 172.6 mg/L), turbidity (1.53 NTU), chlorides (Cl^−^, 10.1 mg/L), iron (Fe, 0.03 mg/L), fluoride (F^−^, 0.25 mg/L), manganese (Mn, 0.001 mg/L), nitrate (${NO}_{3}^{-}$, 4.4 mg/L), sulphate (${SO}_{4}^{2-}$, 37 mg/L), and phosphate (${PO}_{4}^{3-}$, 0.82 mg/L) were all in the limitation of Mexican stand NOM-127-SSA1-2021 [4]. Since there was no arsenic in the selected water, arsenic was added to the stock solution prepared for adsorption tests on synthetic samples. This water was used to prepare the corresponding arsenic solution to the required concentrations by adding sodium arsenite (NaAsO₂, Sigma-Aldrich) and sodium arsenate dibasic heptahydrate (HAsNa_2_O_4_·7H_2_O, Sigma-Aldrich) for studying As(III) and As(V) adsorption processes, respectively. 

The batch adsorption experiment of As(V) using MCs and MXs as adsorbents was conducted to evaluate their adsorption capacities. The effect of solution pH (2–7) on As(V) adsorption was investigated with an initial concentration of 100 µg/L, a dose of 1 g/L, 150 rpm, a contact time of 6 h, and a temperature of 26.2 ± 1 °C. 

Batch adsorption of As(V) using MX4-MX7 and MX8-MX11 was studied with direct sonication at low power output, and indirect sonication at high power output, respectively. The following conditions were used: an initial concentration of As(V) of 200 µg/L, pH of 3, a dose of 2 g/L, 150 rpm, a contact time of 6 h, and a temperature of 26.3 ± 1 °C.

XRF600 was carbonized into pellets and used in this form to test kinetics and isotherms. The kinetic study adsorption using MXRF with As(III) concentrations of 0.025, 0.05, and 0.075 mg/L was conducted at a pH of 3, a dosage of 2 g/L, 150 rpm, a temperature of 26.5 ± 1 °C, and contact time ranging from 10 to 1800 min. The adsorption kinetics of As(III) and As(V) using MXRF600 were carried out under a pH of 3, a dosage of 2 g/L, and initial concentration of As(III) and As(V) solution of 0.514 mg/L and 1.034 mg/L, respectively, with a contact time ranging from 10 to 1440 min.

The conditions for the isotherm adsorption using MXRF600 were as follows: an adsorbent dose of 2 g/L, an initial solution pH of 3.0, 150 rpm, a temperature of 26.4 ± 1 °C, and a contact time of 24 h. The initial concentrations of the As(III) and As(V) solutions were in the range of 0.05 to 1.27 mg/L and 0.12 to 3.0 mg/L, respectively.

The importance of kinetic and equilibrium models of adsorption is described in the mechanisms and dynamics of the adsorption system of adsorbents. Adsorption kinetic models that control the adsorption process of arsenic are related to the adsorbate uptake on the adsorbent with chemisorption. Therefore, the Pseudo First-Order (PFO), Pseudo Second-Order (PSO), and Elovich and Power equations were applied to perform the experimental data in this study. The assumptions of the PFO model are: sorption at localized sites, the energy of adsorption is independent of surface coverage, a saturated monolayer of adsorbates, and the concentration of the adsorbate is constant [73]. The assumptions of the PSO model are similar to those of the PFO model. The PSO kinetic equation typically describes metal ion uptake on activated carbons well, as well as the adsorption of dyes, herbicides, oils, and organic compounds from aqueous solutions [73,74]. The Elovich equation is used to describe the kinetics of a heterogeneous diffusion process [74]. It is a semi-empirical equation that is based on the assumption that the rate of diffusion is controlled by the rate of adsorption onto active sites on the heterogeneity of the surface of the adsorbent.

The Langmuir and Freundlich isotherm models are the most commonly used equilibrium models for determining the relative concentrations of the solute adsorbed onto the solid in the solution [75]. The Langmuir isotherm assumes that a solute is adsorbed onto a homogeneous surface with a finite number of similar active sites, forming a monolayer. The Freundlich isotherm is an empirical model that describes multilayer adsorption.

The equations for kinetic and equilibrium models of adsorption used in this study are listed in Table 7.

The arsenic adsorption process was carried out in a batch reactor system. The effect of contact time and initial concentration of arsenic adsorption was investigated on MXRF and MXRF600. Different kinetic and isotherm adsorption models were analyzed using nonlinear regression analysis with the statistical software R v3.5.

### 4.5. Determination of As(III) and As(V)

The determination of arsenic species was performed using hydride generation atomic absorption spectroscopy (HG-AAS) (Varian; SpectrAA220, Mulgrave, VIC, Australia). To analyze As (III) at trace concentrations, AAS must be combined with the hydride generation (HG) technique with citric-citrate buffer [77]. 

A total of 10 mL of the samples were adjusted to pH 2. In the case of samples of As(V), 1 mL of HCl and 1 mL of potassium iodide and ascorbic acid were added. This was conducted in order to reduce As(V) to As(III). On the other hand, the arsenic (NaAsO_2_) calibration curve was prepared. First, a solution was prepared with 1 mL of arsenic standard and 1 mL of HNO_3_. This solution was then diluted to a concentration of 1 mg/L. From this, the solutions of 0.001, 0.002, 0.004, 0.006, and 0.0075 mg/L were made in 100 mL flasks. A small amount of deionized water was added to each flask, along with 0.1, 0.2, 0.4, 0.6, and 0.75 mL of stock solution, 6 mL of HNO_3_, 10 mL of KI, and 4 mL of HCl. Then, the solutions were calibrated with deionized water. Finally, the solutions were analyzed in a HG-AAS at a wavelength of 193.7 nm.

## Figures and Tables

**Table 1:** The average crystal size of magnetic xerogels varying R/C Molar ratios.

Sample Name	R/C Molar Ratios	Crystal Size Average (nm)	FWHM (β) (Radian)	θ (Radian)
MC1	50	24.52	0.34	35.54
MC2	100	22.94	0.36	35.52
MC4	200	25.88	0.32	35.53

**Table 2:** Textural parameters, pH_pzc_, and IEP of xerogel and Fe_3_O_4_-monolithic resorcinol-formaldehyde xerogels.

Samples	Molar Ratio of R/C	Area BET (m^2^/g)	Total Pore Volume (cm^3^/g)	Average Pore Diameter (nm)	pH_pzc_	IEP
RFX	200	399.19	0.517	5.23	2.99	2.74
MX1	200	472.41	0.842	7.57	4.54	3.09
MC1	50	365.93	0.255	2.79	6.63	3.40
MC2	100	545.09	0.549	4.03	6.12	3.59
MC4	200	529.47	0.683	5.16	4.35	3.70

**Table 3:** Fe_3_O_4_-Monolithic resorcinol-formaldehyde xerogels prepared with direct sonication with low power output varying M/R ratios ranging from 0.03 to 0.2, resulting in different Fe content.

Molar Ratios	Direct Ultrasonic-Assisted Synthesis				
	MX3	MX4	MX5	MX6	MX7
R/W	0.04				
R/C	200				
R/F	0.5				
M/R	0.03	0.05	0.1	0.15	0.2
Fe content (w%)	3.48	5.62	9.29	13.39	13.13
Solids content (*w*/*v*%)	19.59	20.37	22.29	24.23	26.16

**Table 4:** Fe_3_O_4_-Monolithic resorcinol-formaldehyde xerogels prepared with indirect sonication with high power output varying M/R ratios ranging from 0.03 to 0.15.

Molar Ratios	Indirect Ultrasonic-Assisted Synthesis			
	MX8	MX9	MX10	MX11
R/W	0.05			
R/C	200			
R/F	0.5			
M/R	0.03	0.05	0.1	0.15
Fe content (w%)	3.41	5.82	11.59	16.09
Solids content (*w*/*v*%)	23.04	23.95	26.22	28.48

**Table 5:** Kinetic parameters and error indices of Pseudo First-Order, Pseudo Second-Order, Elovich, and Power Equation for As(III) and As(V) removal using MXRF600.

Adsorption	Pseudo First-Order				Pseudo Second-Order			
	q_t_ (µg/g)	k_1_	R²	RMSE	q_t_ (µg/g)	k_2_	R²	RMSE
As(III)	129.68	0.153	0.374	9.900	134.03	0.002	0.575	8.161
As(V)	230.55	0.147	0.446	17.09	238.00	0.001	0.600	14.51
**Adsorption**	**Elovich Equation**				**Power Equation**			
	**α (µg/g min)**	**β (g/µg)**	**R²**	**RMSE**	**a**	**b**	**R²**	**RMSE**
As(III)	808,122.16	0.129	0.830	5.166	93.36	0.062	0.842	4.979
As(V)	2,288,305.88	0.075	0.807	10.07	166.34	0.061	0.822	9.681

**Table 6:** Isotherm parameters and correlation coefficients for As(III) and As(V) adsorption on MXRF600.

Adsorption	Langmuir				Freundlich			
	q_max_ (µg/g)	K_L_ (L/µg)	R²	RMSE	K_F_ ((µg/g)(L/µg)^1/n^)	n	R²	RMSE
As(III)	694.3	1.527	0.897	3.865	502.8	1.346	0.894	3.903
As(V)	1720.3	0.641	0.901	9.220	655.7	1.338	0.899	9.309

**Table 7:** Equation of kinetic and isotherm models of adsorption.

Kinetic Models	Non-Linear Equations	References
Pseudo First-Order	$q_{t}=q_{e}\left(1-exp\left({-k}_{1}\;t\right)\right)$	[74,75]
Pseudo Second-Order	$q_{t}=\frac{{q_{e}}^{2}{\;k}_{2\;}t}{1+k_{2}\;q_{e\;}t}$	[74,75]
Elovich Equation	$q_{t}=\frac{1}{\beta }\ln\left(1+\alpha \beta t\right)$	[76]
Power Equation	$q_{t}={at}^{b}$	[76]
**Isotherm Models**	**Non-Linear Equations**	**References**
Langmuir	$q_{e}=\frac{K_{L}q_{m}C_{e}}{\left(1+K_{L}C_{e}\right)}$	[74]
Freundlich	$q_{e}$ $=K_{F}{C_{e}}^{\frac{1}{n}}$	[74]

*q_t_* and *q_e_* are the amount of adsorbate adsorbed at time *t* (mg/g) and the equilibrium adsorption capacity (mg/g), respectively. *k_1_* is the PFO rate constant (min^−1^), and *k_2_* is the PSO rate constant (min^−1^), respectively. *t* is the contact time (min). α is the initial adsorption rate (mg/g min), *β* is related to surface coverage (g/mg), and *a* and *b* are constants. *C_e_* is the equilibrium concentration of adsorbate in solution (mg/L). *q_m_* is the maximum adsorption capacity (mg/g). *K_L_* is the Langmuir constant that is related to the adsorption energy (L/mg). *K_F_* and *n* are Freundlich constants that measure the adsorption capacity ((mg/g)(L/mg)1/n) and intensity, respectively.

## Data Availability

Not applicable.
